# Genomic organization and gene expression of the multiple globins in Atlantic cod: conservation of globin-flanking genes in chordates infers the origin of the vertebrate globin clusters

**DOI:** 10.1186/1471-2148-10-315

**Published:** 2010-10-20

**Authors:** Ola F Wetten, Alexander J Nederbragt, Robert C Wilson, Kjetill S Jakobsen, Rolf B Edvardsen, Øivind Andersen

**Affiliations:** 1 Department of Animal and Aquacultural Sciences, University of Life Sciences, P.O. Box 5003, N-1430 Aas, Norway; 2 Department of Natural Sciences and Technology, Hedmark University College, P.O. Box 4010 Bedriftsenteret, N-2306 Hamar, Norway; 3 Centre for Ecological and Evolutionary Synthesis (CEES), Department of Biology, University of Oslo, P.O. Box 1066 Blindern, N-0316 Oslo, Norway; 4 Institute of Marine Research, P.O. Box 1870 Nordnes, N-5817 Bergen, Norway; 5 Nofima Marine, P.O. Box 5010, N-1430 Aas, Norway

## Abstract

**Background:**

The vertebrate globin genes encoding the α- and β-subunits of the tetrameric hemoglobins are clustered at two unlinked loci. The highly conserved linear order of the genes flanking the hemoglobins provides a strong anchor for inferring common ancestry of the globin clusters. In fish, the number of *α-β-*linked globin genes varies considerably between different sublineages and seems to be related to prevailing physico-chemical conditions. Draft sequences of the Atlantic cod genome enabled us to determine the genomic organization of the globin repertoire in this marine species that copes with fluctuating environments of the temperate and Arctic regions.

**Results:**

The Atlantic cod genome was shown to contain 14 globin genes, including nine hemoglobin genes organized in two unlinked clusters designated *β5-α1-β1-α4 *and *β3-β4-α2-α3-β2*. The diverged cod hemoglobin genes displayed different expression levels in adult fish, and tetrameric hemoglobins with or without a Root effect were predicted. The novel finding of maternally inherited hemoglobin mRNAs is consistent with a potential role played by fish hemoglobins in the non-specific immune response. *In silico *analysis of the six teleost genomes available showed that the two *α-β *globin clusters are flanked by paralogs of five duplicated genes, in agreement with the proposed teleost-specific duplication of the ancestral vertebrate globin cluster. Screening the genome of extant urochordate and cephalochordate species for conserved globin-flanking genes revealed linkage of *RHBDF1, MPG *and *ARHGAP17 *to globin genes in the tunicate *Ciona intestinalis*, while these genes together with *LCMT *are closely positioned in amphioxus (*Branchiostoma floridae*), but seem to be unlinked to the multiple globin genes identified in this species.

**Conclusion:**

The plasticity of Atlantic cod to variable environmental conditions probably involves the expression of multiple globins with potentially different properties. The interspecific difference in number of fish hemoglobin genes contrasts with the highly conserved synteny of the flanking genes. The proximity of globin-flanking genes in the tunicate and amphioxus genomes resembles the *RHBDF1-MPG-α-globin-ARHGAP17-LCMT *linked genes in man and chicken. We hypothesize that the fusion of the three chordate linkage groups 3, 15 and 17 more than 800 MYA led to the ancestral vertebrate globin cluster during a geological period of increased atmospheric oxygen content.

## Background

Hemoglobin plays a critical role in both terrestrial and aquatic animals by transporting oxygen from the respiratory surface to the inner organs. The functional complexity and evolutionary adaptation of this heme-containing molecule to different environments has therefore attracted researchers for more than a half-century. In jawed vertebrates, or gnathostomes, the hemoglobin tetramer consists of two pairs of α- and β-globins, which probably arose by duplication of a single primordial globin gene about 500-570 million years ago (MYA) [1,2]. Whereas α- and β-globin genes are juxtaposed in teleost fish, birds and mammals are characterized by unlinked clusters of α- and β-globin genes, which in mammals are arranged in the order of their expression during ontogeny [3,4]. Based on the conservation of the globin-flanking genes, including *MPG *and *c16orf35*, all gnathostomes examined share a common globin cluster referred to as the MC locus [5] corresponding to the α-globin cluster in placental mammals and chicken. Silencing of the β genes in the ancestral MC-α-β cluster has apparently also occurred in non-amniotic species, such as pufferfish, whereas a single β-like ϖ-globin is retained in the α cluster of marsupials and monotremes [6-8]. The teleost-specific genome duplication event 350-400 MYA probably gave rise to the second fish α-β globin cluster flanked by *ARHGAP17*, *LCMT *and *AQP8 *[5,8]. It should be noted that this LA locus lacks globin genes in tetrapods, but is positioned on the α-containing chromosome 16 and 14 in man and chicken, respectively [5]. The amniotic β-globin cluster is thought to have originated from the transposition of a β gene copy into a region of olfactory receptor genes in their ancestor [8-10].

In contrast to the linked α-β globin pairs identified in *Xenopus*, the fish α-β pairs are commonly organized head-to-head or tail-to-tail with respect to transcriptional polarity [11-16]. These configurations probably arose from an inversion of one of the paired α-β genes in an ancestral ray-finned fish, thus resembling the reported case of gene inversion within the human β-globin cluster [17]. The structural and functional diversity of the multiple hemoglobins in teleosts strongly indicates that they have experienced a major evolutionary pressure to execute their oxygen-transporting function under highly variable physico-chemical conditions [18-20]. The selective forces have apparently resulted in the loss of hemoglobin genes in the white-blooded Antarctic icefishes (*Channichthyidae*) to reduce the blood viscosity at stable subzero temperatures [21-23].

The genomic organization of the fish α-β globin clusters has only been investigated in the model species pufferfish, zebrafish and medaka [5,6,8,10,15,24]. Atlantic cod is a marine cold water species being widely distributed from the sea surface to depths of 600 m in the Arctic and temperate regions of the North Atlantic Ocean, including the low saline Baltic Sea. Adaptation of the different cod populations to the varying physico-chemical conditions seems to involve hemoglobins with highly pH-sensitive oxygen affinities (Root effect) to adjust the swimming bladder to variable pressure during vertical migrations [25,26], together with the novel feature of expressing polymorphic variants with different oxygen-binding properties [27]. A variable number of cod hemoglobin genes and allelic variants have been reported in Norwegian, Icelandic and Canadian populations [27-29]. Here, we screened the draft cod genome [30] and identified nine α- and β-globin genes, which are organized in two unlinked clusters flanked by highly conserved syntenic regions. We document close linkage between the conserved globin-flanking genes in extant cephalochordate and urochordate species, and hypothesize that the fusion of three chordate chromosomes formed the ancestral vertebrate globin cluster more than 800 MYA.

## Results

### Identification of cod globin clusters

**Figure 1:**
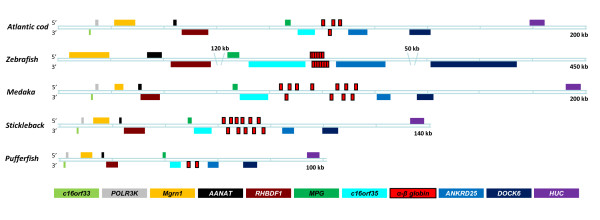
**Conserved synteny at the MC locus in teleosts**. Genomic organization of the Atlantic cod *β5-α1-β1-α4 *globin cluster compared with the orthologous locus in zebrafish, medaka, stickleback and pufferfish. Omitted regions in the zebrafish loci are shown. The boxed genes are shown above or below the doubled line to indicate rightward and leftward transcriptional direction, respectively. The color codes for gene names are shown below.

PCR primers were designed and employed to identify α-β-linked globin genes from genomic DNA, but this strategy resulted only in the amplification of the head-to-head organized *α1*-*β1 *pair (Figure 1). We therefore screened an Atlantic cod Bacterial Artificial Chromosome (BAC) library for α- and β-globin genes. Pyrosequencing of two positive BAC clones respectively resulted in 33,889 and 32,029 reads, which were assembled into 60 (BAC1) and 46 contigs (BAC2). Multiple hemoglobin genes and conserved globin-flanking genes were identified by performing BLAST searches of the contigs using the pufferfish globin loci as query sequences. Finally, the most updated draft sequences from the cod genome project (http://www.codgenome.no) were screened to confirm the gene sequences identified in the BAC clones, and the genomic organization of the hemoglobin loci was determined. The presented sequence information therefore represents the north-east Arctic population of Atlantic cod.

### Cod MC locus

**Figure 2:**
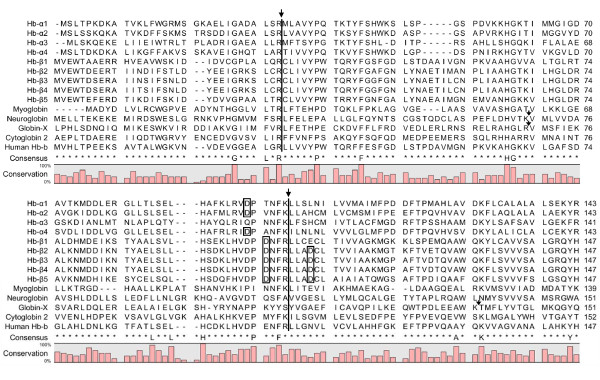
**Sequence alignment of the Atlantic cod α-β globins, myoglobin, neuroglobin globin-X and cytoglobin 2**. The sequences are based on the draft genome of the northeast Arctic population of Atlantic cod. Human β-globin is included for comparison. The alignment was optimized by omitting the N-and/or C-terminal sequences of the non-hemoglobins, and numbers refer to the residues presented. The consensus sequence shows residues with > 80% identity. Putative residues required for Root effect are boxed. GenBank accession numbers: α1 (ACJ66341), α2 (ACJ66342), α3 (ACV69832), α4 (ACV69835), β1 (ACV69840), β2 (ACJ66344), β3 (ACJ66345), β4 (ACJ66346), β5 (ACV69854). Introns are indicated by arrows.

Four hemoglobin genes designated *β5*, *α1*, *β1 *and *α4 *were identified within a 7-kb region of a scaffold spanning 1.7 Mb in the draft cod genome (Figure 1). The hemoglobin genes show the characteristic structure of three exons and two introns encoding the predicted α- and β-globins of 143 and 147 amino acids (aa), respectively (Figure 2). The paired α-β genes are organized tail-to-tail (*β5-α1*), head-to-head (*α1-β1*) or tail-to-head (*β1-α4*), and the *α1 *gene is transcribed in the opposite direction of the others. Seven conserved genes, c*16orf33*, *POLR3K*, *Mgrn1*, *AANAT*, *RHBDF1*, *MPG *and *c16orf35*, were identified within a 90 kb region leftwards of the *β5-α1-β1-α4 *cluster, while the rightwards flanking region of 80 kb harbors *ANKRD25*, *DOCK6 *and *HuC *(Figure 1). A single major regulatory element (MRE; YGCTGASTCAY) was identified as a reversed motif (ATGACTCAGCA) in intron 5 of *RHBDF1 *close to a putative GATA binding site. Whereas paired MREs are located at this position in other vertebrates examined, a second single MRE motif was found in intron 9 of the cod *Mgrn1 *gene. In zebrafish, two additional *Mgrn1 *genes (ENSARG00000018347, ENSDARG00000057481) are also linked to the LA globin locus.

### Cod LA locus

**Figure 3:**
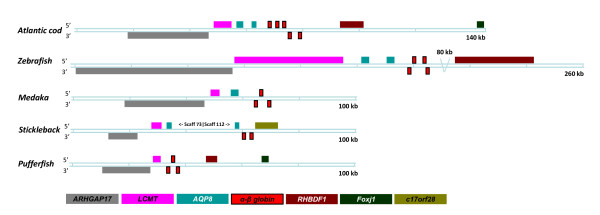
**Conserved synteny at the LA locus in teleosts**. Genomic organization of the Atlantic cod *β3-β4-α2-α3-β2 *globin cluster compared with the counterpart in zebrafish, medaka, stickleback and pufferfish. Further details are as given in Figure 1.

The second cod globin cluster was shown to contain five hemoglobin genes in the order *β3-β4-α2-α3-β2 *positioned within a region of about 12 kb in a scaffold spanning 381 kb (Figure 3). The tail-to-head organized pairs *β4-α2 *and *α3-β2 *are transcribed in opposite directions. The three exons encode the 147-aa long β-globins, while the predicted α3 contains only 141 aa compared to the other α-globins of 143 aa (Figure 2). The globin cluster is flanked on the leftward side by a 70-kb region harboring duplicated *AQP8 *genes similar to the zebrafish locus, and the adjacent *ARHGAP17 *and *LCMT *genes are conserved in the teleosts examined (Figure 3). A *RHBDF1*-like gene is juxtaposed to *FoxJ1 *in the LA locus of only cod and pufferfish, whereas we found a *FoxJ1 *gene coupled to the MC locus in stickleback (ENSGACG00000014879) and zebrafish (ENSDARG00000059545). We also identified paralogs of stickleback *RHBDF1 *(ENSGACG00000004462), *ARHGAP17 *(ENSGACG00000009145) and *FoxJ1 (*ENSGACG00000014879) linked on chromosome 5, which, however, contains no globin genes, whereas an *ARHGAP17 *duplicate is coupled to the MC locus in pufferfish (ENSTING00000017988), zebrafish (ENSDARG00000075341) and medaka (ENSORLG00000009090). A second *ARHGAP17 *gene was also identified in the cod genome, but we presently lack information about any linkage to the globin loci.

### Other cod globin genes

Five additional globin genes encoding myoglobin, neuroglobin, globin-X and two cytoglobins were identified in the cod genome (Figure 2). The gene encoding the predicted cod myoglobin of 145 aa is organized as the α-β globins, while neuroglobin and globin-X of 159 and 197 aa, respectively, are encoded by four and five exons. The three exons of the cytoglobin-2 gene encode a protein of 202 aa, while the draft genome sequences contained only a partial cytoglobin-1 gene. The four α-globins are less similar (35-67% identity) than the five β-globins (57-99%) of which β2, β3 and β4 show high sequence identity. The α-globins share only 25-33% identity with the β-globins, compared to sequence identities of about 20% between the cod α-β globins and the other globins, except for the very low similarity with globin-X. Despite this low overall identity, highly conserved positions were identified throughout the aligned sequences, including human β-globin (Figure 2). Rare mutations in almost all these positions have been reported to affect the functionality of human hemoglobin [31], and suggest the importance of these residues for the proper structure and/or function of different oxygen-binding molecules in diverse vertebrate species.

### Globin gene mapping and expression

The cod α-β globin clusters were mapped to different linkage groups by genotyping multiple single nucleotide polymorphic (SNP) markers, including the globin SNPs underlying the Metβ1Val and Thrα2Ile polymorphisms [27]. The segregation of the SNPs in full-sib cod families localized the MC and LA loci to linkage groups 17 and 16, respectively, among the total of 24 linkage groups [32].

**Figure 4:**
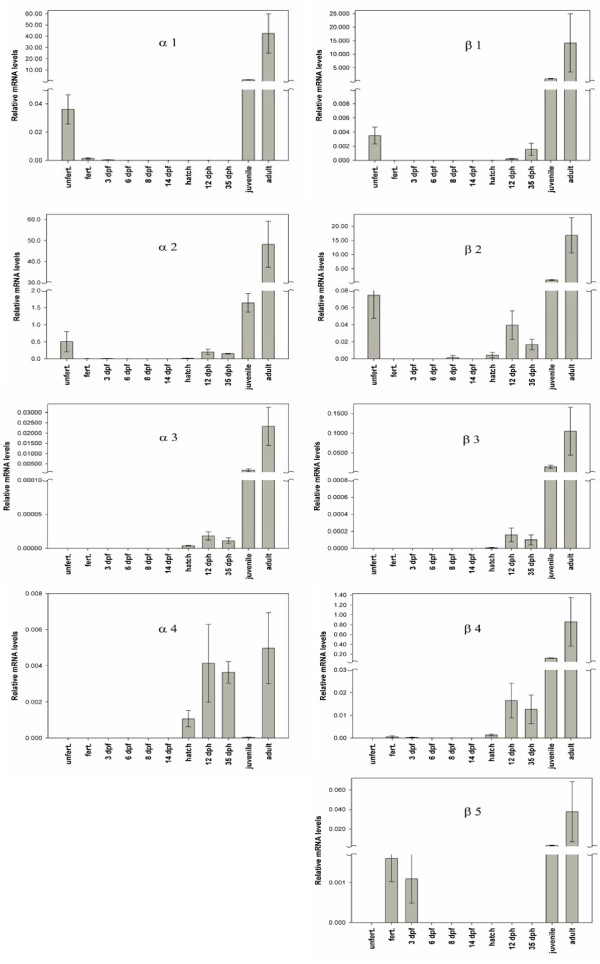
**Real-time PCR analysis of globin gene expression in Atlantic cod**. The globin mRNA levels are presented relative to the level of ubiquitin mRNA at each developmental stage examined. The juvenile and adult expression profiles include spleen and blood mRNAs, respectively. dpf, days post fertilization; dph, days post hatching.

**Figure 5:**
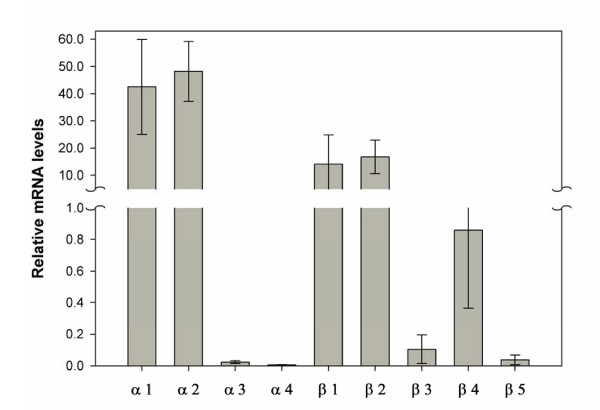
**Comparison of globin mRNA levels quantified in adult Atlantic cod**. See Figure 4 for details.

The nine α-β globin genes were shown to be transcriptionally active by quantifying the mRNA levels throughout the life cycle of Atlantic cod using real-time qPCR (Figure 4). Intriguingly, *α1*, *α2*, *β1 *and *β2 *mRNAs were identified in unfertilized eggs, whereas fertilized eggs and early embryos contained mainly the *β5 *transcript. The later stages of embryogenesis showed very low hemoglobin mRNA levels prior to the larval expression of several α- and β-globin genes, and all hemoglobin genes were expressed in the juvenile and adult fish. Abundant expression of α*1*, *α2*, *β1 *and *β2 *was measured in the adult fish, while the other genes showed low mRNA levels (Figure 5).

## Discussion

The Atlantic cod genome was shown to harbor altogether nine α- and β-globin genes organized in two unlinked clusters similar to the other teleost genomes available. The expression of many hemoglobin genes in adult cod is consistent with the multiple tetrameric hemoglobin types and subtypes identified by gel electrophoresis of blood proteins [33,34]. The cod hemoglobin repertoire is further extended by the polymorphic α1, β1, β3 and β4 globins [27,29] of which the functionally different variants of β1 are differentially distributed in cod populations [27,35,36]. The dominant expression of *α1*, *α2*, *β1 *and *β2 *in adult fish is in agreement with the isolation of three major tetramers designated Hb1, Hb2 and Hb3, which comprise different combinations of these four subunits [37]. The tetrameric Hb3 (α1-α1-β2-β2) was shown to exhibit a marked Root effect of importance for the delivery of oxygen to the swim bladder for neutral buoyancy and to the retina for enhanced visual acuity via the highly specialized vascular structures [25,38]. The structural basis for this extreme acid-induced reduction in oxygen affinity is far from understood, but the putative key residues, including Asp95α, Asp99β and Asp101β [39,40], are conserved in the cod hemoglobins, except for β1 and α3. We therefore suggest that the β1-containing Hb1 tetramer (α1-α1-β1-β1) has no Root effect and might function as an emergency oxygen supplier when fish exercise vigorously.

The detection of hemoglobin mRNAs in unfertilized cod eggs is the first evidence of maternally inherited α-β globins, while Vlecken et al. [41] recently reported maternal transfer of myoglobin mRNA in zebrafish. The function of these oxygen-binding molecules in the early fish embryo is uncertain, as aerobic processes have been shown to continue in the zebrafish embryo after functional ablation of hemoglobin [42]. Hemoglobin-derived antimicrobial peptides expressed in the fish epithelium have been suggested to play a significant role in the non-specific immune response [43], together with maternally transferred transcripts encoding lysozyme and cathelicidin [44]. The very low embryonic expression of globin genes is consistent with the transparent hemolymph flowing through the heart, which starts contracting after embryogenesis is two-thirds completed [45]. Thus, the early larval expression of hemoglobins probably represents the initial stage of hemoglobin oxygen binding and coincides with gill development. The embryonic expression of *β5 *and the dominant mRNA levels of *α4 *at hatching are in agreement with the phylogenetic analysis grouping these genes together with other fish globins expressed in embryonic stages [29].

**Figure 6:**
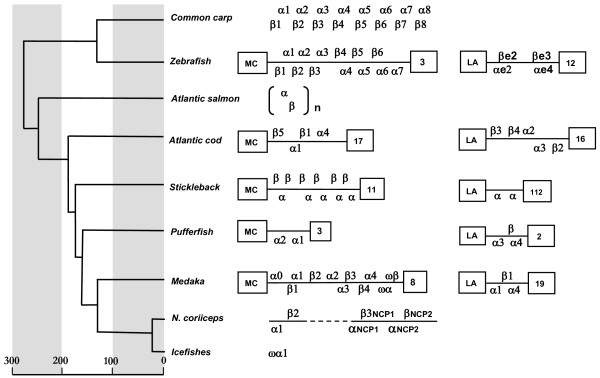
**Comparison of the α-β globin genes in the MC and LA loci of diverse teleosts**. Linkage between globin genes is represented by solid line (broken line indicates uncertainty). The genes are transcribed in the rightward (upper) or leftward (lower) direction. The linkage groups are numbered. The number of paired α-β genes in salmon is unknown (n). The estimated divergence times (MYA) are based on mitochondrial DNA sequences [64].

Duplication and loss of hemoglobin genes have apparently occurred within specific teleost sublineages and have resulted in a variable number of α- and β-globins as summarized in Figure 6. The LA locus comprises from two (stickleback) to five globins (cod), and the phylogenetic analysis of the highly similar β2, β3 and β4 globins in the cod cluster indicated a relatively recent gene duplication event in gadids [29]. Whereas the cod MC locus contains four globins, this cluster harbors up to 13 globins in zebrafish, stickleback and medaka. Maruyama et al. [24] suggested that the latter globin cluster originated from a subcluster duplication, while subsequent gene silencing is evidenced by the ϖβ-ϖα pseudogene pair in medaka. In pufferfish, the MC locus has been reduced to only two α-globins [6], while only remnants of an α-globin gene are found in icefishes inhabiting the cold Polar Ocean saturated with oxygen [46,47]. The metabolic functions are maintained in the hemoglobin-less icefishes by the elevated cardiac output of blood of low viscosity through the highly vascularized gills and skin [48]. Although the Arctic variant of cod β1 and a major β globin component of the pelagic Antarctic teleosts *Pagothenia borchgrevinki *and *Trematonus newnesi *share only 58% sequence identity, similar functional features of these globins were recently hypothesized based on their close position in the PC (principal component) plane in the hydrophobicity analysis of multiple fish globins [49].

**Figure 7:**
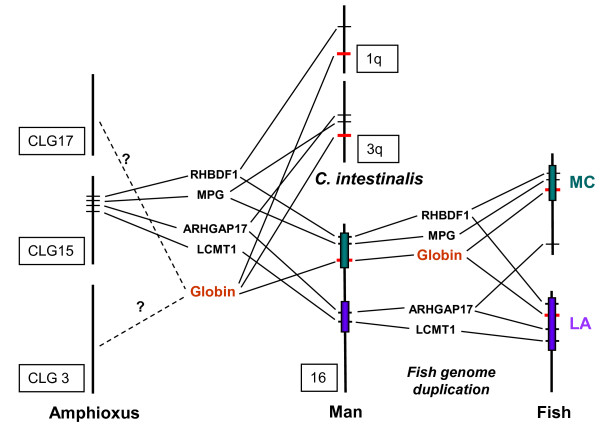
**Proposed model for the evolution of the ancient globin cluster**. Conserved synteny of globin-flanking genes in extant cephalochordate (amphioxus) and urochordate (*C. intestinalis*) species together with the MC (green) and LA (violet) globin loci in man and fish. The fusion of three chordate linkage groups (CLG) to form the homolog of the α-containing human chromosome 16 is based on the reported chromosomal rearrangements of the ancestral chordate genome [53].

The highly conserved linear order of the globin-flanking genes provides a strong anchor for inferring common ancestry of the vertebrate globin clusters. The proposed teleost-specific duplication of an ancient α-β globin cluster implies that paralogs of the flanking genes should still be present in both loci. *In silico *analysis of the teleost genomes available revealed linkage of *RHBDF1*, *ARHGAP17*, *Mgrn1*, *AQP8 *and *FoxJ1 *paralogs to the MC and LA loci in several species. Consistent with these findings, comparative gene mapping of medaka, zebrafish, pufferfish and human genomes demonstrated large conserved syntenic segments in paired fish chromosomes, including the globin-containing pairs of linkage groups 8 and 19 (medaka), 3 and 12 (zebrafish), and 2 and 3 (pufferfish) [50,51] (see Figure 6). Furthermore, we found evidence for the origin of the *RHBDF1-MPG-α-globin-ARHGAP17-LCMT1 *syntenic region in man and chicken by screening the genomes of the tunicate *Ciona intestinalis *and the lancelet *Branchiostoma floridae *(amphioxus). Four *Ciona *globin genes designated *CinHb1-4 *were shown to form a monophyletic group basal to the vertebrate hemoglobin, myoglobin and cytoglobin [52]. We recognized *CinHb3 *(ENSCING00000006495) linked to *MPG *and *ARHGAP17 *on chromosome 3q, while an additional *Ciona *globin gene (ENSCING00000002015) is coupled to *RHBDF1 *on chromosome 1q (Figure 7). In amphioxus, we identified *RHBDF1 *(position 17_000132)*, MPG *(17_000133), *ARHGAP17 *(17_000183) and *LCMT *(17_000184 and 17_000191) on the 4.2-Mb long scaffold 17, which has been localized to the chordate linkage group (CLG) 15 by FISH analysis [53]. We were, however, unable to position any of the multiple globin genes to the 16 scaffolds spanning almost the complete CLG15. Based on conserved chromosomal segments of the amphioxus and human genomes, Putnam et al. [53] reconstructed a total of 17 ancestral CLGs of which CLG3, CLG15 and CLG17 showed syntenic association with the α-containing human chromosome 16. Although we presently lack information about any coupling of the amphioxus globin-like genes to these linkage groups, we propose that the fusion of CLG15 to CLG3 and CLG17 resulted in the linkage of the *RHBDF1-MPG-ARHGAP17-LCMT *region to globin gene(s) as illustrated in Figure 7. The identification of remnants of this globin linkage in the *Ciona *genome indicates that the proposed chromosomal rearrangement occurred prior to the divergence of the vertebrates and urochordates about 800 MYA [54]. Thus, the formation of this syntenic region seems to have coincided with a period of Earth history characterized by a rise in atmospheric oxygen from 0.02-0.04 atm 850 MYA to present day levels of 0.2 atm 540 MYA [55]. The increased oxygen content would be expected to have a strong impact on the regulation and structure of H_2_S-binding globins. In sulfide-rich environments, the unusual sulfide-binding function is found in annelid globins containing key cysteine residues, which are absent in annelid globins from sulfide-free environments [56]. Concomitant with increased atmospheric oxygen, the role of globins as oxygen scavengers would probably be lost in oxygen-tolerant organisms to function as oxygen-transporting hemoglobins. Based on the close phylogenetic relation of cyclostome hemoglobins to gnathostome cytoglobins, the ancestors of cyclostome and gnathostome vertebrates were recently stated to have independently invented erythroid-specific oxygen-transporting hemoglobins about 450-600 MYA [57]. The transcriptional regulation of the hemoglobins in extant vertebrates involves both proximal promoters and distant enhancers [58]. In mouse erythroid cells, the active *α1 *and *α2 *genes are in close spatial proximity of the flanking *RHBDF1*, *MPG *and *c16orf35*, including the *cis-*regulatory MREs, as the result of erythroid-specific changes in the chromatin conformation [59]. The chromosomal rearrangements forming this highly conserved syntenic region seem to have occurred more than 800 MYA, and we therefore propose that the molecular mechanism underlying the oxygen-dependent regulation of globin expression evolved prior to the structural changes in the duplicated ancestral globins.

## Conclusions

In contrast to the low number of globin genes reported in Antarctic teleosts [23], the adaptation of Atlantic cod to fluctuating environmental conditions probably involved the evolution of multiple globins with potentially different oxygen binding properties. The unlinked globin pairs *α1-β1 *and *α2-β2 *are abundantly expressed in the adult fish and form three major hemoglobin tetramers with different Root effect. The identification of paralogous genes in the flanking regions of the two globin clusters in diverse teleosts supports the proposed teleost-specific duplication of the vertebrate globin cluster. Based on the conserved synteny of globin-flanking genes in extant urochordate and cephalochordate species, we hypothesize that the ancestral globin cluster contained both the MC and LA loci, and was formed by the fusion of three chordate chromosomes. We propose that these chromosomal rearrangements facilitated the transcriptional regulation of globin synthesis to cope with increased atmospheric oxygen content about 850 MYA. Thus, these regulatory changes probably preceded the convergent evolution of different ancestral globins to function as erythroid-specific oxygen transporting hemoglobins.

## Methods

### Identification of globin clusters

#### PCR

Forward and reverse PCR primers were designed to amplify pairs of α-β genes using cod globin gene sequences available in GenBank (Table. 1). PCR was performed under standard conditions (Applied Biosystems 2720 thermal cycler) using genomic DNA as template (Qiagen DNeasy blood & tissue kit). The amplified products were ligated into the pGEM^®^-T easy vector (Promega) and sequenced in both directions (Applied Biosystems 3130xl genetic analyzer).

**Table 1:** PCR primers for amplification of cod α1-β1 gene pair and for screening BAC library.

Gene	Name	Sequence (5' to 3')
*α1-β1*	A1-B1F	GCAAATTGTTCAAGTTATTCCCCCTAAC
	A1-B1R	TAAAGACTGACCTGCAACGCGAGTGGT
*α1*	A1-bacF	CAGACCAAGACTTACTTCAGCC
	A1-bacR	GCTCGCTCAGAGTGAGAAGAC
*α2*	A2-bacF	CCGATGATATCGGAGCTGAGG
	A2-bacR	CTAAGGCTGAGGAGTCCTCC
*β1*	B1-bacF	ATGGTTGAATGGACAGCTGC
	B1-bacR	GTCGACGTGCAGTTTCTC
*β2*	B2-bacF	TGGACAGATAGTGAGCGCG
	B2-bacR	AGTGGAGCAGAGACAGCTC

#### BAC library screening

A cod BAC library consisting of 92,000 clones with average insert size of 125 kb was screened for globin genes by PCR using gene specific primers (Table 2) on pools and super-pools of BAC clones. Positive BAC clones were purified (NucleoBond BAC 100), and sequenced using the 454 GS FLX instrumentation at the Norwegian Sequencing Center (http://www.sequencing.uio.no). The resulting reads were assembled using Newbler v. 2.0 (gsAssembler) [60], using default settings and filtering of the reads against contaminating *E. coli *genomic sequences. The pufferfish globin loci (AY016023, *Sphoeroides nephelus; *AY016024, *Takifugu rubripes*) were utilized as query sequences in BLAST searches of the assembled contigs.

**Table 2:** Primers for real-time qPCR, amplification efficiency (%) and amplicon size (bp).

Gene	Name	Sequence (5' to 3')	Efficiency	Size
*α1*	A1F	GACTTACTTCAGCCACTGGAAGAGCCT C	96	153
	A1R	TTGAAGGCGTGCAGCTCGCTCAGAG		
*α2*	A2F	GTCCTATTTCTCTCACTGGAAGGACGCG	85	153
	A2R	ATGAACGCGTGCAGCTCGCTAAGGC		
*α3*	A3F	CACATCATACCCTGGCACCAAGAC	95	172
	A3R	CTGGTAGGCGTGGTAGGTTTGAAGAG		
*α4*	A4F	TTCTCCCACTGGAAAGACCTCGG	70	138
	A4R	ATGGAGCTCACTGAGCTCGAGAAG		
*β1 allele A*	B1FA	TTATGGGAAACCCCAAGGTGGCCAA	91	131
	B1R	GTGCAGTTTCTCGGAGTGCAGCACGC		
*β1 allele B*	B1FB	TTGTGGGAAACCCCAAGGTGGCTGC	98	131
	B1R	GTGCAGTTTCTCGGAGTGCAGCACGC		
*β2*	B2F	CCTGTACAATGCAGAGACCATCATGGC	84	151
	B2R	GTGCAGCTTGTCAGAGTGGAGCAGAG		
*β3*	B3F	ACAGATAGTGAGCGCGCCATCATTAA	86	176
	B34R	GCGGCGATCAGGGGGTTGCACAG		
*β4*	B4F	ACAGATAGTGAGCGCGCCATCATTAC	95	176
	B34R	GCGGCGATCAGGGGGTTGCACAG		
*β5*	B5F	GTGGACTCGGAGGTACTTTGGAAAC	89	168
	B5R	TGCAGCTGACTGAGCTCGCAATAG		
*Ubiquitin*	UbiF	GGCCGCAAAGATGCAGAT	81	69
	UbiR	CTGGGCTCGACCTCAAGAGT		

#### Cod genome BLAST

The Atlantic cod genome project (http://www.codgenome.no) is based on the genome sequences of the north-east Arctic cod population. Scaffold sequences harboring globin genes were identified among the assemblies of the cod genome project [30] using the BLAST search tool at http://www.bioportal.uio.no. Annotation of genes located on the scaffolds was completed based on results from TBLASTN searches of known protein sequences from related species, using the bioinformatics software CLC genomics workbench (CLC bio).

#### Chordate genome BLAST

Conserved globin and globin-flanking genes were identified in cephalochordate and urochordate species by BLAST searching the genomes of *Branchiostoma floridae *(version 1.0, http://genome.jgi-psf.org/Brafl1/Brafl1.home.html) and *Ciona intestinalis *(release 43, http://www.ensembl.org/Ciona_intestinalis/Info/Index).

### Real-time qPCR

#### Fish

Spleen and blood were sampled from juvenile (n = 5) and adult (n = 12) fish kept at the National Cod Breeding Centre (Kraknes, Tromsø, Norway) and the University of Bergen, respectively. Sexually mature fish were hand-stripped, and eggs were fertilized *in vitro*. The incubation of embryos and feeding of larvae were carried out as described [44]. Sampling of unfertilized eggs, fertilized eggs and larvae was performed during 10 weeks. All samples were rapidly submerged in RNAlater (Ambion, Austin, TX, USA) and incubated at 4°C overnight, then stored at -20°C.

#### RNA isolation and cDNA synthesis

5-10 eggs/embryos or 3-5 larvae were pooled and homogenized in 1.5 ml microcentrifuge tubes containing lysis buffer (Qiagen RNeasy mini kit) using a plastic pestle. After centrifugation through a QiaShredder column (Qiagen, Hilden, Germany), RNA was isolated according to the manufacturer's protocol (Qiagen RNeasy mini kit), and followed by the recommended on-column DNase treatment. The Qiagen RNeasy mini kit was also used for the spleen and blood samples from juvenile and adult fish, respectively. cDNA was synthesized from 1 μg total RNA using TaqMan^® ^Reverse Transcription Reagents (Applied Biosystems) and oligo-dT primer in 20 μl reactions using the conditions of: 25°C for 10 min, 48°C for 30 min and 95°C for 5 min. Primers used for real-time qPCR were adopted from Borza et al. [29] for the globins, while *ubiquitin *primers were taken from Olsvik et al. [61] (Table 2). For the *β1 *gene, two allele-specific primer sets were used on all samples, and relative expression was calculated dependent on the actual genotype of each sample. Ten-fold dilution series were prepared to generate standard curves, and PCR efficiencies and relative quantification results were calculated according to Ståhlberg et al. [62] using ubiquitin as the reference transcript [63]. Cycling parameters were 50°C for 2 min, 95°C for 10 min, 40 cycles of 95°C for 15 sec, 61°C for 1 min, including a final dissociation stage to yield melting curves. Reactions of 25 μl consisted of 12.5 μl 2× Power SYBR^®^Green PCR Master Mix (Applied Biosystems), 0.5 μl each of sense and antisense primers (10 μM) and 11.5 μl of 50× diluted cDNA.

## List of abbreviations

MPG: N-methylpurine-DNA glycosylase; ARHGAP17: Rho GTPase activating protein 17; RHBDF1: rhomboid 5 homolog 1; LCMT: leucine carboxyl methyltransferase; c16orf35: human chromosome 16 open reading frame 35; POLR3K: DNA-directed RNA polymerase III subunit RPC10; Mgrn1: mahogunin Ring Finger 1; AANAT: arylalkylamine N-acetyltransferase; DOCK: dedicator of cytokinesis; ANKRD: ankyrin repeat domain; AQP: aquaporin; FoxJ1: fork head J1; PC: principial component; FISH: fluorescence *in situ *hybridization; CLG: chordate linkage group; dpf: days post fertilization; dph: days post hatching.

## Authors' contributions

OFW carried out the majority of the analyses. AJN performed the sequencing and assembly of the reads. RW participated in the real-time PCR analysis. KSJ participated in the design of the study and the sequencing. RBE screened the BAC library. ØA conceived and designed the study, and wrote the manuscript. All authors critically read the manuscript drafts and approved the final version of the manuscript.
